# Swallowing Disorders after Oral Cavity and Pharyngolaryngeal Surgery and Role of Imaging

**DOI:** 10.1155/2017/7592034

**Published:** 2017-03-22

**Authors:** Caterina Giannitto, Lorenzo Preda, Valeria Zurlo, Luigi Funicelli, Mohssen Ansarin, Salvatore Di Pietro, Massimo Bellomi

**Affiliations:** ^1^Division of Radiology, European Institute of Oncology, Via Ripamonti 435, Milan, Italy; ^2^Department of Clinical-Surgical, Diagnostic and Pediatric Sciences, University of Pavia and Diagnostic Imaging Unit, National Center of Oncology Hadrontherapy (CNAO) Pavia, Privata Strada Campeggi 55, 27100 Pavia, Italy; ^3^Division of Otolaryngology and Head and Neck Surgery, European Institute of Oncology, Via Ripamonti 435, Milan, Italy; ^4^School of Radiology, University of Milan, Via Morandi 30, 20097 San Donato Milanese, Italy

## Abstract

Head and neck squamous cell carcinoma is the sixth most common cancer diagnosed worldwide and the eighth most common cause of cancer death. Malignant tumors of the oral cavity, oropharynx, and larynx can be treated by surgical resection or radiotheraphy with or without chemotheraphy and have a profound impact on quality of life functions, including swallowing. When surgery is the chosen treatment modality, the patient may experience swallowing impairment in the oral and pharyngeal phases of deglutition. A videofluoroscopic study of swallow enables the morphodynamics of the pharyngeal-esophageal tract to be accurately examined in patients with prior surgery. These features allow an accurate tracking of the various phases of swallowing in real time, identifying the presence of functional disorders and of complications during the short- and long-term postoperative recovery. The role of imaging is fundamental for the therapist to plan rehabilitation. In this paper, the authors aim to describe the videofluoroscopic study of swallow protocol and related swallowing impairment findings in consideration of different types of surgery.

## 1. Introduction

Head and neck squamous cell carcinoma (HNSCC) is the sixth most common cancer diagnosed worldwide and the eighth most common cause of cancer death [1].

Malignant tumors of the oral cavity, oropharynx, and larynx can be treated by surgical resection or radiotheraphy with or without chemotheraphy and have a profound impact on quality of life functions, including swallowing.

When surgery is the chosen treatment modality, the patient may experience swallowing impairment in the oral and pharyngeal phases of deglutition.

Effects of various surgical procedures for oropharyngeal cancer include a reduced range of oral tongue motion, reduced oral tongue coordination, reduced posterior movement of tongue base, with prolonged bolus transit through the oropharynx, and abnormal swallowing efficiency [2–7]. Approximately one-sixth of patients with prior laryngectomy present dysphagia related to the morphofunctional changes carried out on the pharynx during surgery [8–16]. A videofluoroscopic study of swallow enables the morphodynamics of the pharyngeal-esophageal tract to be accurately examined in patients with prior surgery. In particular, the digital system enables the analysis of individual images “frame by frame” and the dynamic sequence immediately after the acquisition, which allows an immediate assessment of the findings. These features allow an accurate tracking of the various phases of swallowing in real time, identifying the presence of functional disorders and of complications during the short- and long-term postoperative recovery [16].

The direct observation of the effectiveness of swallowing while varying a number of factors (density of the barium, position of the head) can provide information regarding certain compensatory mechanisms [16].

So, the role of imaging is fundamental for the therapist to plan rehabilitation.

In this paper, the authors aim to describe the videofluoroscopic study of swallow protocol and related swallowing impairment findings in consideration of different types of surgery.

## 2. The Videofluoroscopic Swallow Study Protocol

In order to reliably compare studies between patients and within patients pre- and postintervention, it is essential to perform every investigation in a standardized and systematic manner.

The authors suggest Logemann's protocol [17] of modified barium swallow procedure, recording the digital fluoroscopic images permanently on a DVD film.

All studies must be performed by a licensed radiology technician and a speech language pathologist and reviewed by a specialized radiologist.

The ability to record the entire study with a frame rate higher than 30 frames per second (fps) and being able to review the film in a frame-by-frame manner are essential for precise interpretation and analysis. Studies obtained at capture rates lower than 30 fps may miss significant pathology [18].

All examinations may be reviewed by an interdisciplinary panel that includes a speech language pathologist, a surgeon, and a radiologist.

Patients should be weighed, measured, and given the validated ten-item Eating Assessment Tool (EAT-10) prior to the administration of barium [18] (Table 1). The EAT-10 documents the level of baseline disability and helps in monitoring treatment efficacy. The patient is positioned upright with the fluoroscopy unit in the lateral position. The patient's head position is neutral and facing forward. The boundaries of the fluoroscopic field in the lateral view are the lips anteriorly, nasopharynx superiorly, cervical spine posteriorly, and cervical esophagus inferiorly (seventh cervical vertebra inferiorly). The boundaries of the fluoroscopy field in the anteroposterior (AP) view are the walls of the pharynx laterally and the nasopharynx [17].

The study protocol should proceed in a stepwise fashion. While viewed in the lateral plane, the patient should be given two swallows of each of the following materials. Liquid barium in measured amounts is the first material used.

Liquid is the first material administered, even if the patient is known to aspirate, because it is usually best to define the reason for aspiration and the amount of aspiration during the first swallows and because liquid is least likely to block airway. After two 1 ml swallows are completed, two 3 ml liquid swallows should be given. Then, two 5 ml swallows are given. If no aspiration occurs at 5 ml, 10 ml should be given. Second, swallows of thicker foods should be given diluting barium sulfate nectar from semisolid to semiliquid formulation. The last material used is a cookie with a light coating of barium. In this case, the patient is told to go ahead and swallow as soon as he or she has completed chewing [19].

When the desired number of swallows of various materials has been completed in the lateral plane, the patient should be turned to be viewed in the anteroposterior plane, evaluating the symmetry of swallow. Only swallows of those food consistencies that were most difficult for the patient should be repeated in this view [19].

It is useful to structure findings during examination in a swallowing worksheet. The authors suggest the use of Logemann's worksheet [19] for each consistency of food in the lateral view and in the anteroposterior view only for those food consistencies that were most difficult for the patient (Tables 2(a)–2(d)).

In case of penetration and aspiration, the use of penetration and aspiration scale (Table 3) is mandatory. In fact, this eight-point scale assesses depth of bolus passage into the airway and the patient's response to the bolus [20].

Compensatory strategies could redirect and/or improve the flow and direction of food and may change the dimensions of the pharynx, and so giving better airway protection without increasing the effort or work for the patient during the swallow.  Table 4 presents the postures which have current evidence for their use and their rationale [21].

## 3. Swallowing Disorders Related to Specific Surgical Resection and Reconstruction Technique: What the Radiologist Should Know

To understand the swallowing disorders after surgery, the radiologist should know the exact nature and extent of the resection of the tumor and the exact nature of the reconstruction.

Now, the authors show you the postoperative pictures of oral cavity and pharynx during the examination in relation to the different types of surgery.

## 4. Findings after Oral Cavity and Oropharyngeal Surgery

Based on location, size, and extent of the tumor as well as the surgical reconstruction procedure, cancer in the oral cavity can significantly affect the functional outcome [2, 26–28].

According to Groher [27], the removal of less than 50% of a structure involved with swallowing will not interfere or seriously impact swallowing and the resulting swallowing difficulties are temporary [28].

**Figure 1:**
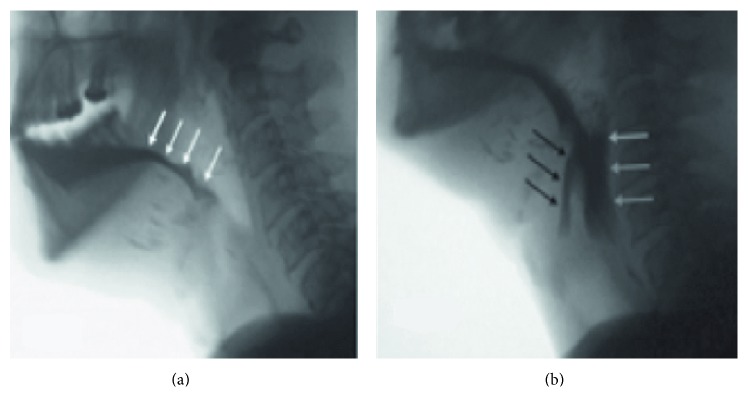
Lateral fluoroscopic view of a 49-year-old man who has undergone a near-total glossectomy for advanced head and neck cancer. A small amount of tongue is seen. The patient has poor oral bolus control and early loss into the oropharynx ((a) white arrows). He has lost his ability to pull the hyoid and the larynx up and forward to open the upper esophageal sphincter resulting in pharyngeal dysphagia and food remaining in pharynx (white arrows) with penetration just over arytenoid complex, remaining above the vocal folds (black arrows). This represents a penetration and aspiration score of 3 (b).

When more than 50% of the tongue is resected, lingual propulsion and control of material in the mouth are severely reduced, because the contact between the remaining tongue segment and the palate is lost (Figure 1(a)).

The area excised and surgical procedure are prognostic indicators of the resultant dysphagia, especially, in cases of base of tongue and arytenoid cartilage resections [29].

If neural control and some tongue movement were preserved, swallowing could not be very compromised [30].

When the resection of tissue is so large that there is no sufficient tissue remaining to permit primary closure, the surgeon may need to borrow tissue from another area of the body, by means of a flap or graft [19].

This may interfere with the neural control, by the use of a nonsensate flap [31] or by hypoglossal nerve involvement [32], with resulting disorders of the bolus passage through the oropharynx and problems with chewing, controlling food in the mouth, and initiating swallowing.

When total glossectomy is required, swallowing impairment is related with the mandible and the floor of the mouth muscle resection.

In fact, when glossectomy is combined with anterior mandible resection and/or removal of these muscles, the patient loses the ability to pull the hyoid and the larynx up and forward to open the upper esophageal sphincter leading to pharyngeal dysphagia and food remaining in the pyriform sinus and in the pharynx [19, 33] (Figure 1(b)).

The removal of the upper margin of the mandible and a portion of the floor of the mouth with closure of the defect with a flap has few functional changes. The same resection by suturing the tongue into surgical defect will present severe difficulties with lost lingual control and propulsion of the bolus and mastication [19]. If sacrifice of the hyomandibular constrictors is required, the protective tilting action of the larynx is lost and there may be a risk of aspiration.

Considering the location, if the tumor site is in the posterior oral cavity and oropharynx, this can be a bad prognostic indicator [7]. In fact, surgical procedure can minimize the tongue base to posterior pharyngeal wall contact with reduced pressure generation and delayed initiation of the swallow. In many cases, this results in aspiration before the swallow or pharyngeal stasis and postswallow aspiration.

**Figure 2:**
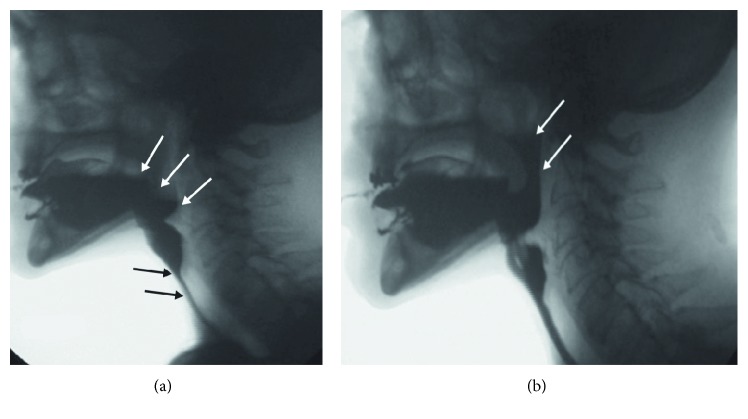
Lateral fluoroscopic view of a 74-year-old man who has undergone a previous laryngectomy and subsequent resection of the left posterior tongue and left tonsillar region. The patient has poor oral bolus control and early loss into the neopharynx ((a), white arrows). Black arrows show a narrowing in the neopharynx with dysfunction of reconstructed cricopharyngeal junction and residue throughout the neopharynx. (b) Palatopharyngeal valve dysfunction and reflux of contrast (white arrows) between the soft palate and the posterior pharyngeal wall.

Combined resections of the tongue and hard palate change the bolus passage into the pharynx. Combined resections of the soft palate and tonsillar pillars alter the bolus transport through the oral cavity and pharynx with nasopharyngeal reflux and pharyngeal stasis too (Figure 2).

Resection of cancer in the ipopharynx can result in significant swallowing impairment, because of alterations of muscular contraction that may cause missed pharyngeal clearance, with pharyngeal residue and risk of penetration and aspiration [34].

When bilateral neck dissections are needed, this results in poor swallowing unless the superior laryngeal nerve, hyoid bone, and epiglottis remain intact.

## 5. Findings after Partial and Total Laryngectomy

The laryngeal complex serves two critical functions during swallowing. First, the larynx elevates and moves anteriorly under the tongue base to move it from the path of the bolus and to assist in cricopharyngeal sphincter opening.

Second, it protects the airway from aspiration by closing at three levels: the epiglottis, false vocal folds, and true vocal folds. Any surgery that compromises this closure, especially that of the true vocal folds, is likely to result in aspiration during the swallow.

Tipping the patient's head forward to push the epiglottis more posteriorly could avoid the airway entrance [19]. If there is still aspiration with the chin down, head rotation to the operated side may improve airway protection.

If a supraglottic laryngectomy is required, the whole supraglottis is resected, including the pre-epiglottic space and the upper half of the thyroid cartilage. Inferiorly, the resection encompasses the petiole of the epiglottis, down to the anterior commissure, and then the ventricular folds. Posteriorly, the limit of resection passes in front of the arytenoids, sectioning the ventricular and aryepiglottic folds. Superiorly, the incision transects the valleculae along the posterior aspect of the hyoid bone. Larynx reconstruction is accomplished by a thyrohyoidopexy. In individual cases, resection of the hyoid bone may be necessary. In these cases, the pexy will approximate the inferior thyroid and the base of the tongue. The laryngeal elevation is thus damaged. If the hyoid bone is removed, laryngeal suspension and elevation are damaged. If a laryngeal suspension procedure is performed during reconstruction, laryngeal elevation is improved and swallowing is safety enhanced [35]. Horizontal supraglottic laryngectomy could be extended to the base of the tongue or to the pyriform sinus.

In contrast to total laryngectomy, in partial laryngectomy, the communication between the airways and the digestive tract remains, but the excision of some of the structures which serve to prevent the bolus passing into the trachea (epiglottis, arytenoids, or vocal cords) explains why in these patients the most common postoperative complication is aspiration.

To eliminate aspiration, these patients should occlude the airway retracting the tongue base to make contact with the anteriorly tilting arytenoid [19]. So the tongue base makes complete contact with the posterior pharyngeal wall. If complete contact is not made, there will be residue in the pharynx that falls directly into the airway.

In supracricoid laryngectomy (SCPL) for transglottic tumors with glottic and supraglottic involvement and minimal extension to the infraglottis, the hyoid bone, the cricoid cartilage, and at least one arytenoid are preserved, thus maintaining the possibility of functional reconstruction. There are two forms of laryngeal reconstruction: cricohyoidopexy (CHP), in which the cricoid cartilage is placed closer to the hyoid bone (for supraglottic tumors that are nonresectable by supraglottic laryngectomy), and cricohyoidoepiglottopexy (CHEP), in which the epiglottis is maintained and its lower portion is included in the suture that approximates the cricoid to the hyoid bone (for glottic region tumors) [36]. The differences between the various subtypes of supracricoid laryngectomy are related to the amount of supraglottis removed and their extension, if any, to include one arytenoid.

The key functional outcomes are airway, phonation, and swallowing without aspiration. Phonation and swallowing depend on the arytenoids being able to tilt forwards and make contact with the base of the tongue; to breathe, the arytenoids tilt posteriorly to open the airway. An intact cricoarytenoid unit is critical for function. Sacrificing one unit increases the chance of disabling aspiration in the cases where the epiglottis is resected.

**Figure 3:**
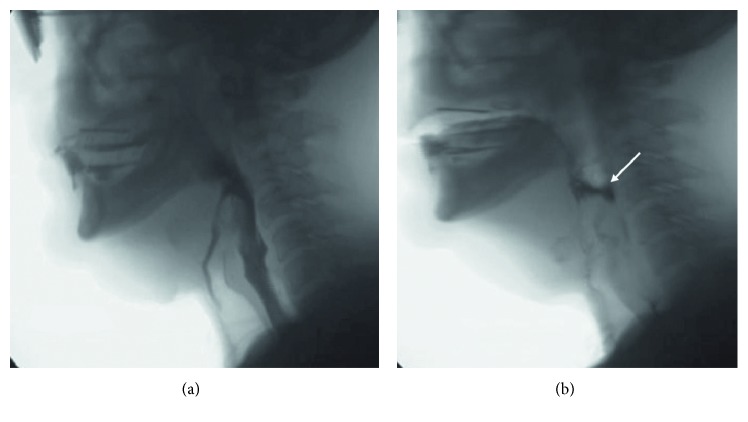
Lateral fluoroscopic view of a 51-year-old man who has undergone a supracricoid laryngectomy. The bolus enters the airway and passes below the vocal folds, and no effort is made to eject. This represents a penetration and aspiration score of 8 (a). (b) Cricopharyngeal dysfunction with residue in the pharynx, white arrow.

SCPL initially results in severe swallowing dysfunction, most notably aspiration, but permits the eventual return to oral nutrition for most patients [37–39] (Figure 3).

Supratracheal laryngectomy entails the resection of the entire supraglottic, glottic, and part of the subglottic sites, sparing both or at least one functioning cricoarytenoid unit.

A standard total laryngectomy involves dissection of the larynx from above the hyoid bone to below the cricoid cartilage. The suprahyoid muscles are dissected from the hyoid bone [40].

The thyropharyngeus muscle is shaved off the thyroid cartilage, and the cricopharyngeus muscle is removed from the cricoid cartilage.

All of these muscles are preserved and later reconstructed to form the pharyngoesophageal segment (PES) which allows for optimum voice and swallowing function [40]. Once the larynx is removed, the opened trachea and pharynx remain.

The exposed upper part of the trachea is secured to an opening in the neck to form a stoma, through which the patient will breathe. The open pharynx then is closed. Provided that enough thyropharingeal and cricopharyngeal muscle remains, these are used as a second level of closure over the repaired pharynx [40].

This second layer of closure inwardly compresses the repaired pharyngeal tissue. At rest, this is seen on videofluoroscopy as a closed narrow area and is referred to as the pharyngoesophageal (PE) or reconstructed segment [40].

The reconstructed pharynx after laryngectomy is referred to as a neopharynx.

The ability of the PES to dilate, coupled with the power created at the base of the tongue and the length of PES which remains open, dictates the patient's ability to swallow a variety of food consistencies. In the pharyngeal phase, the reconstruction of suprahyoids by suturing them onto the superior margin of the repaired thyroglossus pull on the reconstructed segment, helping to lift the pharynx which in turn helps to relax the reconstructed thyropharyngeus opening entrance to the esophagus. The bolus under reduced pressure from the tongue base and pharyngeal wall contraction exerts pressure from above. This together with gravity moves the bolus onward.

72% of laryngectomy patients reported symptoms of dysphagia [41].

Before commencing the videofluoroscopic examination of these patients, a metal marker should be placed to the right side of the stoma to indicate stoma level. Patient should be placed in a lateral oblique position to observe the hypopharingeal area and upper esophagus [40].

In the anteroposterior view, following the bolus as it passes from the neopharynx through the cricopharyngeus enables the study of esophageal swallow. It should also provide a careful assessment of the entire esophagus as far as the oesophagogastric junction for the possibility of low benign stenosis, as shown by Gibbons et al. [9] and for a gastroesophageal reflux.

Classic findings after laryngectomy are pseudodiverticulum and fistula.

Pseudodiverticulum is a mucosalized pouch at the base of the tongue, separated from the remaining pharynx by a posterior tissue band (pseudoepiglottis). A large diverticulum may obstruct bolus flow. The incidence of fistulas reported in the literature is approximately 6% and they usually develop in the immediate postoperative period [42].

**Figure 4:**
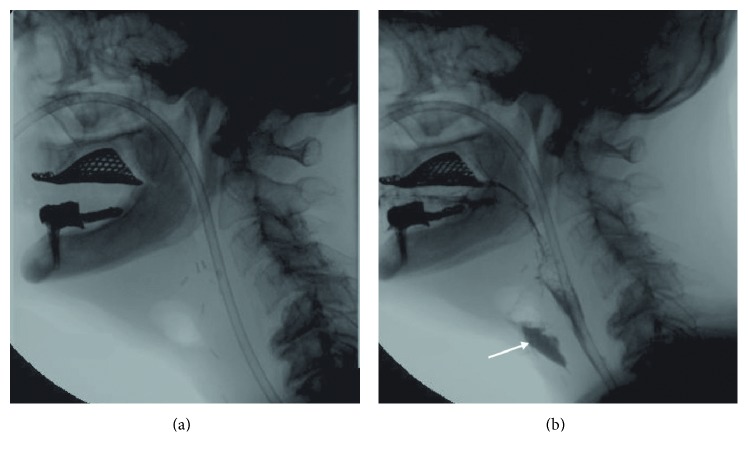
Lateral fluoroscopic views of a 67-year-old man who has undergone a total laryngectomy with fistula. A radiolucent area in front of the neopharynx suggests fistula (a). In (b), an extraluminal collection of liquid barium (white arrow) confirms the presence of fistula.

They generally originate from the anterior wall in relation to the suture at the lingual base, from the borders of the transplanted musculocutaneous flaps, and from the lateral wall and the pyriformis sinuses. In an attempt to reach the cutaneous layer, they often end blindly in an extraluminal collection [12, 42] (Figure 4).

## 6. Conclusion

Swallowing mechanisms are different in relation to the various types of surgery. The more the radiologist knows of the highly diverse anatomical and functional changes involved in the surgery, the more precise the analysis is of the swallowing function at baseline and the evaluation of effectiveness of treatment strategies carried out to improve the patient's swallow.

The best rehabilitation goals in head and neck cancer patients can be attained only by a team of specialists, including a radiologist, a surgeon, a speech-language pathologist, and a deglutition therapist who can plan and initiate the appropriate therapy, monitoring the improvements over time.

## Figures and Tables

**Table 1:** Eating Assessment Tool (EAT-10) [24].

To what extent are the following scenarios problematic for you?	0 = no problem, 4 = severe problem				
(1) My swallowing problem has caused me to lose weight.	0	1	2	3	4
(2) My swallowing problem interferes with my ability to go out for meals.	0	1	2	3	4
(3) Swallowing liquids takes extra effort.	0	1	2	3	4
(4) Swallowing solids takes extra effort.	0	1	2	3	4
(5) Swallowing pills takes extra effort.	0	1	2	3	4
(6) Swallowing is painful.	0	1	2	3	4
(7) The pleasure of eating is affected by my swallowing.	0	1	2	3	4
(8) When I swallow, food sticks in my throat.	0	1	2	3	4
(9) I cough when I eat.	0	1	2	3	4
(10) Swallowing is stressful.	0	1	2	3	4
	Total EAT-10				

**(a):** 

Lateral view	Consistency of food	
Preparation to swallow	Amount of bolus	Possible swallowing disorders
Cannot hold food in mouth anteriorly		Reduced lip closure
Cannot form bolus		Reduced tongue movement rage or coordination
Cannot hold bolus—premature bolus loss Aspiration (%) before swallow		Reduced tongue shaping/coordination; reduced velar movements
Material falls into anterior sulcus		Reduced labial tension
Materials falls into lateral sulcus		Reduced buccal tension
Abnormal hold position		Tongue thrust; reduced tongue control
Posture introduced		

**(b):** 

Lateral view	Consistency of food	
Oral phase	Amount of bolus	Possible swallowing disorders
Delayed oral onset of swallow		Apraxia of swallow; reduced oral sensation
Searching tongue movements		Apraxia of swallow
Tongues moves forward to start to swallow		Tongue thrust
Residue in anterior sulcus		Reduced labial tension; reduced lingual control
Residue in lateral sulcus		Reduced buccal tension
Residue on floor of mouth sulcus		Reduced tongue shaping or coordination
Residue in midtongue depression		Tongue scarring
Residue on tongue		Reduced tongue movement and strength
Disturbed lingual contraction		Disorganized A-P tongue
Incomplete tongue-palate contact		Reduced tongue elevation
Residue on hard palate		Reduced tongue elevation and strength
Reduced A-P tongue movement		Reduced A-P lingual coordination
Uncontrolled bolus/premature swallow		Reduced tongue control; reduced linguavelar seal
Aspiration (%) before swallow		Reduced tongue control
Piecemeal deglutition		
Oral transit time		
Posture/treatment introduced		

**(c):** 

Lateral view	Consistency of food	
Pharyngeal phase	Amount of bolus	Possible swallowing disorders
Nasal penetration		Reduced velopharyngeal closure
Pseudoepiglottis (total laryngectomy)		
Coating on pharyngeal walls after swallow		Reduced pharyngeal contraction
Vallecular residue (%) after swallow Aspiration of this (%) after swallow		Reduced tongue base posterior movement
Coating in depression on pharyngeal walls Aspiration of this (%) after swallow		Scar tissue; pharyngeal pouch
Residue at top of airway		Reduced laryngeal elevation
Aspiration of this (%) after swallow		
Penetration into airway entrance Aspiration of this (%) after swallow		Reduced laryngeal elevation/reduced closure of airway entrance
Reduced laryngeal closure Aspiration of this (%) after swallow		Reduced closure of airway entrance
Aspiration during swallow		Reduced laryngeal closure
Residue in both pyriform sinuses Aspiration of this (%) after swallow		Reduced laryngeal anterior notion, cricopharyngeal dysfunction, stricture
Residue throughout the pharynx Aspiration of this (%) after swallow		Generalized reduced pression during swallow
Pharyngeal transit time		
Posture introduced		
*Cervical esophageal phase*		
Esophageal-to-pharyngeal backflow		
Tracheoesophageal fistula		
Other		

**(d):** 

Anteroposterior view	Consistency of food	
Pharyngeal phase	Amount of bolus	Possible swallowing disorders
Unilateral vallecular residue		Unilateral dysfunction of tongue base
Residue in one pyriform sinus		Unilateral dysfunction of pharynx
Reduced laryngeal movement medially		Reduced adduction
Unequal height of vocal folds		
Posture introduced		

**Table 3:** Penetration and aspiration scale (PAS).

Score	Description
1	Material does not enter the airway
2	Material enters the airway, remains above the vocal folds, and is ejected from the airway
3	Material enters the airway, remains above the vocal folds, and is not ejected from the airway
4	Material enters the airway, contacts the vocal folds, and is ejected from the airway
5	Material enters the airway, contacts the vocal folds, and is not ejected from the airway
6	Material enters the airway, passes below the vocal folds, and is ejected into the larynx or out of the airway
7	Material enters the airway, passes below the vocal folds, and is not ejected from the trachea despite effort
8	Material enters the airway, passes below the vocal folds, and no effort is made to eject

**Table 4:** Postures used for eliminating aspiration or residue, the disorders they are designed to address, and the rationale for their use [19, 21].

Disorders on videofluoroscopic swallow	Posture applied	Rationale
Inefficient oral transit	Head back	Gravity to clear oral cavity [22]
Delay in triggering the pharyngeal swallow	Chin down	Widens valleculae, stop bolus entering airways [23]
Reduced posterior tongue base movement	Chin down	Pushes the tongue back toward pharyngeal wall [24]
Unilateral vocal fold palsy, surgical removal of vocal cord (aspiration during swallow)	Head rotated to affect side	Directs bolus down stronger side, improves vocal cold closure [22, 25]
Reduced closure of laryngeal entrance and vocal folds (aspiration during swallow)	Chin down Head rotated to affect side	Improves protective position of epiglottis, narrows laryngeal entrance [24]
Unilateral pharyngeal palsy	Head rotated to affect side	Directs bolus down stronger side of pharynx [24, 25]
Reduced pharyngeal contraction	Lying down on one side	Eliminating gravity effect on laryngeal residue
Unilateral oral and pharyngeal weakness	Head rotated to damaged side	Directs bolus down stronger side by gravity
Cricopharyngeal dysfunction (residue in pyriform sinuses)	Head rotated	Pulls cricoid cartilage from posterior pharyngeal wall reducing pressure at cricopharyngeal junction
